# Draft genome sequence of *Micromonospora* sp. DSW705 and distribution of biosynthetic gene clusters for depsipeptides bearing 4-amino-2,4-pentadienoate in actinomycetes

**DOI:** 10.1186/s40793-016-0206-2

**Published:** 2016-10-22

**Authors:** Hisayuki Komaki, Natsuko Ichikawa, Akira Hosoyama, Moriyuki Hamada, Enjuro Harunari, Arisa Ishikawa, Yasuhiro Igarashi

**Affiliations:** 1 Biological Resource Center, National Institute of Technology and Evaluation, Chiba, Japan; 2 NBRC, Tokyo, Japan; 3 Biotechnology Research Center and Department of Biotechnology, Toyama Prefectural University, Toyama, Japan

**Keywords:** Actinomycete, BE-43547, *Micromonospora*, Nonribosomal peptide synthetase, Polyketide synthase, Rakicidin, Taxonomy, Vinylamycin

## Abstract

Here, we report the draft genome sequence of *Micromonospora* sp. DSW705 (=NBRC 110037), a producer of antitumor cyclic depsipeptides rakicidins A and B, together with the features of this strain and generation, annotation, and analysis of the genome sequence. The 6.8 Mb genome of *Micromonospora* sp. DSW705 encodes 6,219 putative ORFs, of which 4,846 are assigned with COG categories. The genome harbors at least three type I polyketide synthase (PKS) gene clusters, one nonribosomal peptide synthetase (NRPS) gene clusters, and three hybrid PKS/NRPS gene clusters. A hybrid PKS/NRPS gene cluster encoded in scaffold 2 is responsible for rakicidin synthesis. DNA database search indicated that the biosynthetic gene clusters for depsipeptides bearing 4-amino-2,4-pentadienoate are widely present in taxonomically diverse actinomycetes.

## Introduction

In our screening of antitumor compounds from rare actinomycetes, *Micromonospora* sp. DSW705 collected from deep seawater was found to produce rakicidins A and B. Rakicidins are fifteen-membered cyclic depsipeptides comprising three amino acids and a modified fatty acid. The most intriguing feature of rakicidins is the presence of a rare unusual amino acid, 4-amino-2,4-pentadienoate (APDA) in their cyclic structures, which is present only in a limited range of secondary metabolites of actinomycetes [1–3]. To date, five rakicidin congeners have been reported; rakicidins A, B, and E were isolated from *Micromonospora* *,* and rakicidins C and D from *Streptomyces* [4–7]. Recently, we disclosed the biosynthetic gene (*rak*) cluster for rakicidin D through the genome analysis of *Streptomyces* sp. MWW064 and proposed its biosynthetic pathway (Komaki1 H, Ishikawa A, Ichikawa N, Hosoyama A, Hamada M, Harunari E, Nihira T, Panbangred W, Igarashi Y. Draft genome sequence of *Streptomyces* sp. MWW064 for elucidating the rakicidin biosynthetic pathway. Stand Genomic Sci.), if its volume and pages are determined. In this study, the whole genome shotgun sequencing of *Micromonospora* sp. DSW705 was conducted to assess its potential in secondary metabolism, to identify the biosynthetic genes for rakicidins A and B, and to make a comparative analysis with the gene cluster of rakicidin D in *Streptomyces* sp. MWW064. We here report the draft genome sequence of *Micromonospora* sp. DSW705, together with the taxonomical identification of the strain, description of its genome properties, and annotation of the rakicidin gene cluster. Furthermore, we investigated distribution of the *rak*–like clusters in other bacterial strains to evaluate the gene distribution in taxonomically diverse actinomycetes.

## Organism information

### Classification and features

**Fig. 1:**
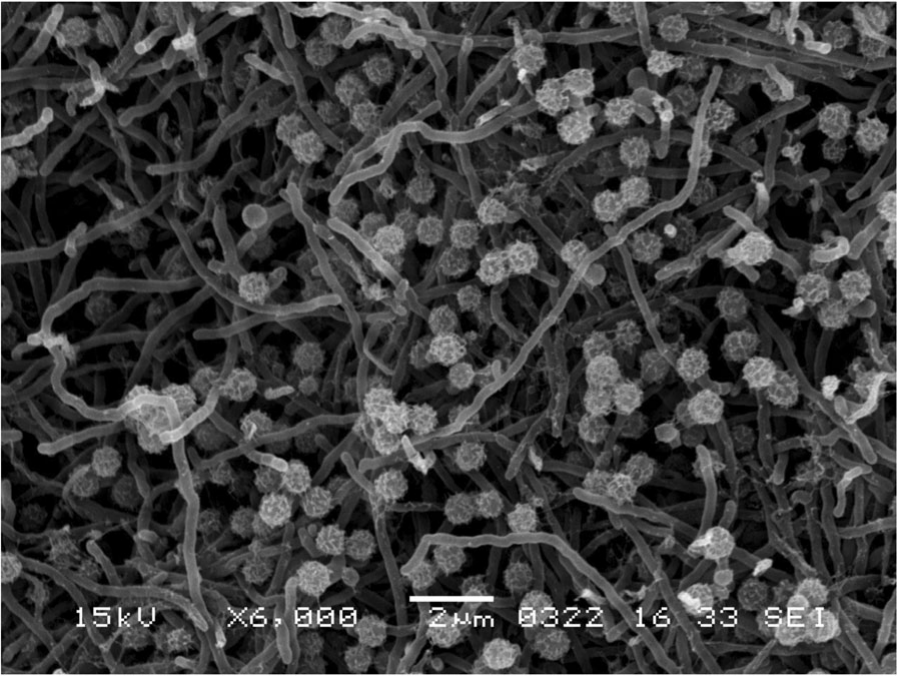
Scanning electron micrograph of *Micromonospora* sp. DSW705 grown on 1/2 ISP 2 agar for 7 days at 28 °C. Bar, 2 μm

**Fig. 2:**
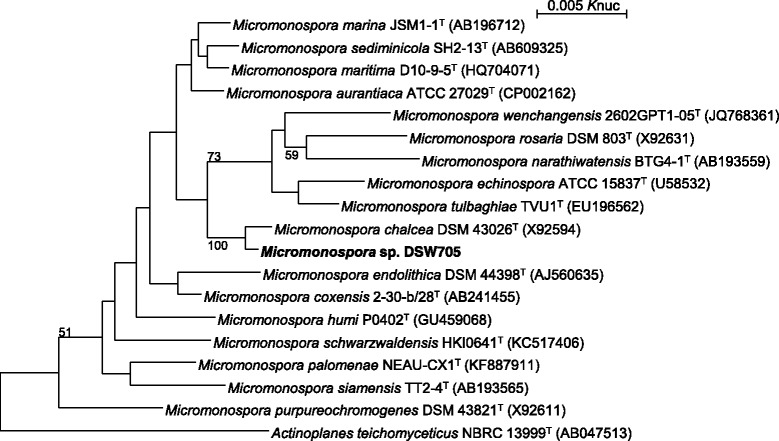
Phylogenetic tree of *Micromonospora* sp. DSW705 and phylogenetically close type strains showing over 98.5 % similarity to strain DSW705 based on 16S rRNA gene sequences. The accession numbers for 16S rRNA genes are shown in parentheses. The tree was reconstructed by the neighbor-joining method [33] using sequences aligned by ClustalX2 [10]. All positions containing gaps were eliminated. The building of the tree also involves a bootstrapping process repeated 1,000 times to generate a majority consensus tree, and only bootstrap values above 50 % are shown at branching points. *Actinoplanes teichomyceticus* NBRC 13999^T^ was used as an outgroup. Bar, 0.005 *K*_nuc_ substitutions per nucleotide position

In the screening of antitumor compounds from rare actinomycetes, *Micromonospora* sp. DSW705 was isolated from deep seawater collected in Toyama Bay, Japan and found to produce BU-4664 L and rakicidins A and B (unpublished). The general feature of this strain is shown in Table 1. This strain grew well on ISP 2 and ISP 4 agars. On ISP 7 agars, the growth was poor. No growth was observed on ISP 5 agar. No aerial mycelia were observed. Substrate mycelium was orange, turning dark brown on sporulation on ISP 2 agar. No diffusible pigment was observed on ISP 2, ISP 3, ISP 4, ISP 5, ISP 6, and ISP 7 agar media. The strain bored single spore on short sporophore. The spores were spherical (0.7–0.8 μm in diameter) with wrinkle surface. A scanning electron micrograph of the strain is shown in Fig. 1. Growth occurred at 20–45 °C (optimum 37 °C) and pH 5–8 (optimum pH 7). Strain DSW705 exhibited growth with 0–3 % (w/v) NaCl (optimum 0 % NaCl). Strain DSW705 utilized arabinose, fructose, glucose, raffinose, sucrose, and xylose for growth. This strain was deposited in the NBRC culture collection with the registration number of NBRC 110037. The genes encoding 16S rRNA were amplified by PCR using two universal primers, 9 F and 1541R. After purification of the PCR product by AMPure (Beckman Coulter), the sequencing was carried out according to an established method [8]. Homology search of the sequence by EzTaxon-e [9] indicated the highest similarity (99.66 %, 1448/1453) to *Micromonospora chalcea* DSM 43026^T^ (X92594) as the closest type strain. A phylogenetic tree was reconstructed using ClustalX2 [10] and NJPlot [11] on the basis of the 16S rRNA gene sequence together with those of taxonomically close type strains showing over 98.5 % similarities. Evolutionary distances were calculated using Kimura’s two-parameter model [12]. The tree has been deposited into TreeBase (http://purl.org/phylo/treebase/phylows/study/TB2:S19405). In the phylogenetic tree, strain DSW705 and *M. chalcea* DSM 43026^T^ (X92594) formed a monophyletic cluster with a bootstrap resampling value of 100 % (Fig. 2).

**Table 1:** Classification and general features of *Micromonospora* sp. DSW705 [15]

MIGS ID	Property	Term	Evidence code^a^
	Classification	Domain *Bacteria*	TAS [22]
		Phylum *Actinobacteria*	TAS [23]
		Class *Actinobacteria*	TAS [24]
		Order *Actinomycetales*	TAS [24–27]
		Suborder *Micromonosporineae*	TAS [24, 27]
		Family *Micromonosporaceae*	TAS [24, 26–29]
		Genus *Micromonospora*	TAS [26, 30]
		Species undetermined	-
		Strain DSW705	IDA
	Gram stain	Not tested, likely positive	NAS
	Cell shape	Branched mycelia	IDA
	Motility	Not reported	
	Sporulation	Sporulating	IDA
	Temperature range	Grows from 20 °C to 45 °C	IDA
	Optimum temperature	37 °C	IDA
	pH range; Optimum	5 to 8; 7	IDA
	Carbon source	Arabinose, fructose, glucose, raffinose, sucrose, xylose	IDA
MIGS-6	Habitat	Sea water	NAS
MIGS-6.3	Salinity	Grows from 0 % to 3 % NaCl	IDA
MIGS-22	Oxygen requirement	Aerobic	IDA
MIGS-15	Biotic relationship	Free-living	IDA
MIGS-14	Pathogenicity	Not reported	
MIGS-4	Geographic location	Toyama Bay, Japan	NAS
MIGS-5	Sample collection	October 10, 2005	NAS
MIGS-4.1	Latitude	Not reported	
MIGS-4.2	Longitude	Not reported	
MIGS-4.4	Altitude	Not reported	

^a^Evidence codes - *IDA* Inferred from Direct Assay, *TAS* Traceable Author Statement (i.e., a direct report exists in the literature), *NAS* Non-traceable Author Statement (i.e., not directly observed for the living, isolated sample, but based on a generally accepted property for the species, or anecdotal evidence). These evidence codes are from the Gene Ontology project [31]

### Chemotaxonomic data

The isomer of diaminopimelic acid in the whole-cell hydrolysate was analyzed according to the method described by Hasegawa et al. [13]. Isoprenoid quinones and cellular fatty acids were analyzed as described previously [14]. The whole-cell hydrolysate of strain DSW705 contained *meso*-diaminopimelic acid as its diagnostic peptidoglycan diamino acid. The predominant menaquinone was identified as MK-10(H_4_); MK-9(H_4_), MK-10(H_2_), and MK-10(H_6_) were also detected as minor components. The major cellular fatty acids were found to be *iso*-C_16:0_, *iso*-C_15:0_ and *anteiso*-C_17:0_.

## Genome sequencing information

### Genome project history

In collaboration between Toyama Prefectural University and NBRC, the organism was selected for genome sequencing to elucidate the rakicidin biosynthetic pathway. The draft genome sequences have been deposited in the INSDC database under the accession number BBVA01000001-BBVA01000024. The project information and its association with MIGS version 2.0 compliance are summarized in Table 2 [15].

**Table 2:** Project information

MIGS ID	Property	Term
MIGS 31	Finishing quality	Improved-high-quality draft
MIGS-28	Libraries used	454 shotgun library, Illumina paired-end library
MIGS 29	Sequencing platforms	454 GS FLX+, Illumina HiSeq1000
MIGS 31.2	Fold coverage	5 ×, 100 ×, respectively
MIGS 30	Assemblers	Newbler v2.6, GenoFinisher
MIGS 32	Gene calling method	Progidal
	Locus tag	MSP03
	Genbank ID	BBVA00000000
	GenBank date of release	April, 2016
	GOLD ID	Not registered
	BioProject	PRJDB3540
MIGS 13	Source material identifier	NBRC 110037
	Project relevance	Industrial

### Growth conditions and genomic DNA preparation

*Micromonospora* sp. DSW705 was deposited in the NBRC culture collection with the registration number of NBRC 110037. The monoisolate of strain DSW705 was grown on a polycarbonate membrane filter (Advantec) on double diluted NBRC 227 agar medium (0.2 % yeast extract, 0.5 % malt extract, 0.2 % glucose, 2 % agar, pH 7.3) at 28 °C. High quality genomic DNA for sequencing was isolated from the mycelia using an EZ1 DNA Tissue Kit and a Bio Robot EZ1 (Qiagen) according to the protocol for extraction of nucleic acid from Gram-positive bacteria. The size, purity, and double-strand DNA concentration of the genomic DNA were measured by pulsed-field gel electrophoresis, ratio of absorbance values at 260 nm and 280 nm, and Quant-iT PicoGreen dsDNA Assay Kit (Life Technologies), respectively, to assess the quality of genomic DNA.

### Genome sequencing and assembly

Shotgun and paired-end libraries were prepared and subsequently sequenced using 454 pyrosequencing technology and HiSeq1000 (Illumina) paired-end technology, respectively (Table 2). The 36 Mb shotgun sequences and 682 Mb paired-end sequences were assembled using Newbler v2.6 and subsequently finished using GenoFinisher [16] to yield 24 scaffolds larger than 500 bp. The N50 was 629,027 bp.

### Genome annotation

Coding sequences were predicted by Prodigal [17] and tRNA-scanSE [18]. The gene functions were annotated by an in-house genome annotation pipeline, and searched for domains related to polyketide synthase (PKS) and nonribosomal peptide synthetase (NRPS) using the SMART and PFAM domain databases. PKS and NRPS gene clusters and their domain organizations were determined as reported previously [8] and using antiSMASH [19]. Substrates of adenylation (A) and acyltransferase (AT) domains were predicted using antiSMASH. BLASTP search against the NCBI nr databases were also used for predicting function of proteins encoded in the *rak* cluster.

## Genome properties

The total size of the genome is 6,795,311 bp and the GC content is 72.9 % (Table 3), similar to other genome-sequenced *Micronomospora* members. Of the total 6,273 genes, 6,219 are protein-coding genes and 54 are RNA genes. The classification of genes into COGs functional categories is shown in Table 4. As for secondary metabolite pathways by modular PKSs and NRPSs, *Micromonospora* sp. DSW705 has at least three hybrid PKS/NRPS gene clusters, three type I PKS gene clusters, and one NRPS gene clusters. According to the assembly line mechanism [20], we predicted the chemical structures which each cluster would synthesize (Table 5), suggesting the potential of *Micromonospora* sp. DSW705 to produce diverse polyketide- and nonribosomal peptide-compounds as secondary metabolites.

**Table 3:** Genome statistics

Attribute	Value	% of Total
Genome size (bp)	6,795,311	100.0
DNA coding (bp)	6,219,133	91.5
DNA G + C (bp)	4,955,456	72.9
DNA scaffolds	24	-
Total genes	6,273	100.0
Protein coding genes	6,219	99.1
RNA genes	54	0.9
Pseudogenes	-	-
Genes in internal clusters	2,376	37.8
Genes with function prediction	3,909	62.3
Genes assigned to COGs	4,846	77.2
Genes with Pfam domains	5,528	84.1
Genes with signal peptides	480	7.7
Genes with transmembrane helices	1,546	24.6
CRISPR repeats	0	-

**Table 4:** Number of genes associated with general COG functional categories

Code	Value	% age	Description
J	234	4.8	Translation, ribosomal structure and biogenesis
A	1	0.02	RNA processing and modification
K	606	12.5	Transcription
L	285	5.9	Replication, recombination and repair
B	2	0.04	Chromatin structure and dynamics
D	63	1.3	Cell cycle control, Cell division, chromosome partitioning
V	125	2.6	Defense mechanisms
T	315	6.5	Signal transduction mechanisms
M	281	5.8	Cell wall/membrane biogenesis
N	37	0.76	Cell motility
U	77	1.6	Intracellular trafficking and secretion
O	174	3.6	Posttranslational modification, protein turnover, chaperones
C	345	7.1	Energy production and conversion
G	475	9.8	Carbohydrate transport and metabolism
E	587	12.1	Amino acid transport and metabolism
F	110	2.2	Nucleotide transport and metabolism
H	221	4.5	Coenzyme transport and metabolism
I	277	5.7	Lipid transport and metabolism
P	344	7.1	Inorganic ion transport and metabolism
Q	282	5.8	Secondary metabolites biosynthesis, transport and catabolism
R	984	20.3	General function prediction only
S	457	9.4	Function unknown
-	1,373	28.3	Not in COGs

The total is based on the total number of protein coding genes in the genome

**Table 5:** Modular PKS and NRPS gene clusters in *Micromonospora* sp. DSW705

Gene cluster	Encoded in	No. of modular PKS and NRPS genes	No. of modules	Backbone of predicted product
*pks/nrps-1* (*rak*)	scaffold 2	6	7	R-C_3_-C_3_^a^-Ser-C_2_-Gly-X
*pks/nrps-2*	scaffold 2	6	6	X-X-X-?-C_2_-Ser
*pks/nrps-3*	scaffold 2	5	6	X-X-?-C_2_-Asn-Ser
*pks-1*	scaffold 2	12	33	R-C_2_-C_3_-C_2_-C_2_-C_2_-C_2_-C_2_-C_4_-C_2_-C_2_-C_2_-C_2_-C_2_-C_2_-C_2_-C_2_-C_2_-C_?_-C_2_-C_3_-C_2_-C_2_-C_3_-C_2_-C_3_-C_3_-C_2_-C_3_-C_3_-C_3_-C_2_-C_2_
*pks-2*	scaffold 5	1	1	C_2_
*pks-3*	scaffold 24	1	1	C_2_
*nrps-1*	scaffold 2	2	2	X-Ala

*R* starter molecule, *C*_*3*_ C_3_ unit derived from methylmalonyl-CoA, *C*_*2*_ C_2_ unit derived from malonyl-CoA, *X* amino acid unpredicted, *?* lack of A domain in the NRPS module, *C*_*4*_ C_4_ unit derived from ethylmalonyl-CoA or methoxymaronyl-CoA, *C*_*?*_ substrate of AT domain was not predicted

^a^Although antiSMASH predicted that the AT domain incorporates malonyl-CoA as the substrate, the signature sequence for substrate determination is not HAFHS for malonyl-CoA but TSSHS likely for methylmaronyl-CoA [32]

## Insights from the genome sequence

### Rakicidin biosynthetic gene cluster in *Micromonospora* sp. DSW705

**Fig. 3:**
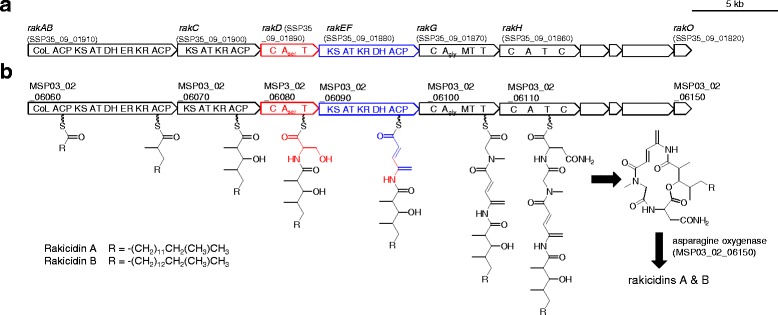
Genetic map of rakicidin biosynthetic gene cluster of (**a**) *Streptomyces* sp. MWW064 and (**b**) *Micromonospora* sp. DSW705 and the biosynthetic mechanism of rakicidins A and B

Our previous study revealed that rakicidin is synthesized by a hybrid PKS/NRPS gene cluster. Its domain organization is shown in Fig. 3a (SIGS-D-16-00018.2). Among the three hybrid PKS/NRPS gene clusters present in the *Micromonospora* sp. DSW705 genome shown in Table 5, only *pks*/*nrps*-1 shows the same domain organization as the *rak* cluster of *Streptomyces* sp. MWW064 (Fig. 3b). Since this gene cluster encodes all the enzymes necessary for assembling the rakicidin core structure, this cluster was confirmed as a *rak* cluster (Table 6). Gene organizations of the clusters for rakicidin D in *Streptomyces* sp. MWW064 (Fig. 3a) and rakicidins A and B in *Micromonospora* sp. DSW705 (Fig. 3b) are essentially identical. Proposed biosynthetic pathway for rakicidins in *Micromonospora* sp. DSW705 is illustrated in Fig. 3b.

**Table 6:** ORFs in the rakicidin-biosynthetic gene cluster of *Micromonospora* sp. DSW705

MSP03_02_ (locus tag)	Size (aa)	Deduced function	Protein homolog [origin]	Identity/similarity (%)	Accession number
06020	1,046	Transcriptional regulator	Transcriptional regulator [*Micromonospora purpureochromogenes*]	95/95	WP_030498969
06030	564	Monooxygenase	Monooxygenase [*Micromonospora purpureochromogenes*]	94/95	WP_030498970
06040	314	Unknown	Hypothetical protein [*Salinispora pacifica*]	66/75	WP_027650590
06050	674	Unknown	LigA protein [*Micromonospora* sp. M42]	99/99	EWM62996
06060	2,944	PKS	Hypothetical protein [*Micromonospora purpureochromogenes*]	95/96	WP_036342114
06070	1,608	PKS	Non-ribosomal peptide synthetase [*Micromonospora* sp. M42]	93/93	EWM63000
06080	1,123	NRPS	Non-ribosomal peptide synthetase [*Micromonospora* sp. M42]	99/100	EWM63002
06090	1,883	PKS	Beta-ketoacyl synthase [*Micromonospora purpureochromogenes*]	97/97	WP_030498975
06100	1,517	NRPS	Hypothetical protein, partial [*Micromonospora purpureochromogenes*]	97/97	WP_036342201
06110	1,563	NRPS	Hypothetical protein [*Micromonospora purpureochromogenes*]	95/95	WP_030498977
06120	570	ABC transporter	Pyoverdine ABC transporter permease/ATP-binding protein [*Micromonospora* sp. M42]	100/100	EWM63008
06130	287	Type-II thioesterase	Gramicidin S biosynthesis protein GrsT [*Micromonospora* sp. M42]	98/98	EWM63009
06140	955	NRPS	Non-ribosomal peptide synthetase [*Micromonospora* sp. M42]	99/99	EWM63010
06150	329	Asparagine oxygenase	Clavaminate synthase [*Micromonospora* sp. M42]	100/100	EWM63011
06160	771	Transporter	Membrane protein mmpL11 [*Micromonospora* sp. M42]	99/99	EWM63012

### Biosynthetic gene clusters for rakicidins and the related compounds in other strains

**Fig. 4:**
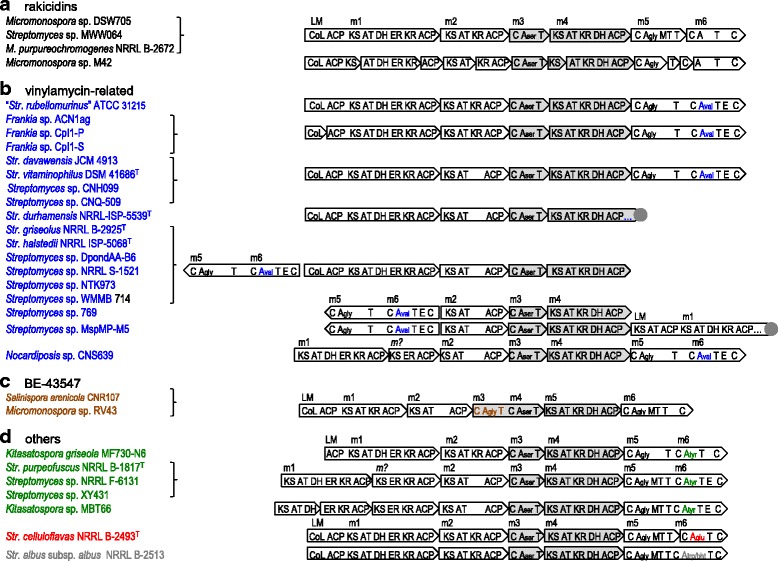
Hybrid PKS/NRPS gene clusters for depsipeptides bearing 4-amino-2,4-pentadienoate (APDA) moieties in published genome sequences of actinomycete strains. Gene clusters for rakicidins (**a**), vinylamycin-related compounds (**b**), BE-43547 (**c**), and others (**d**). NRPS and PKS genes for the synthesis of APDAs are shaded in light gray. Terminals of scaffold sequences are shown in dark gray circles. Locus tag numbers of ORFs in this figure are as follows: *Micromonospora purpureochromogenes* NRRL B-2672, IH31_RS0100575 to IH31_RS0100600; *Micromonospora* sp. M42, MCBG_00130 to MCBG_00140; “*Streptomyces rubellomurinus*” ATCC 31215, VM95_RS28100 to VM95_RS28120; *Frankia* sp. ACN1ag, UK82_23055 to UK82_23085; *Frankia* sp. CpI1-P, FF86_101835 to FF86_101841; *Frankia* sp. CpI1-S, FF36_02633 to FF36_02639; *Streptomyces davawensis* JCM 4913, BN159_0686 to BN159_0681; *Streptomyces vitaminophilus* DSM 41686^T^, A3IG_RS0122990 to A3IG_RS0122970; *Streptomyces* sp. CNH099, B121_RS0112700 to B121_RS0112685 and B121_RS37950; *Streptomyces* sp. CNQ-509, AA958_29290 to AA958_29325; *Streptomyces durhamensis* NRRL-ISP-5539^T^, IO33_RS0129710 to IO33_RS0129695; *Streptomyces griseolus* NRRL B-2925^T^, IH14_RS0112325 to IH14_RS0112355; *Streptomyces halstedii* NRRL ISP-5068^T^, IG73_RS0111725 to IG73_RS0111755; *Streptomyces* sp. DpondAA-B6, K379_RS0125155 to K379_RS0125185; *Streptomyces* sp. NRRL S-1521, ADL30_05665 to ADL30_05635; *Streptomyces* sp. NTK973, DT87_RS01535 to DT87_RS01505; *Streptomyces* sp. WMMB 714, H181_RS01075 to H181_RS01105; *Streptomyces* sp. 769, GZL_RS00255 to GZL_RS00285; *Streptomyces* sp. MspMP-M5, B073_RS0123900 to B073_RS40860; *Nocardiposis* sp. CNS639, G011_RS0119410 to G011_RS0119385; *Salinispora arenicola* CNR107, F583_RS01000000127215 to F583_RS01000000127205; *Micromonospora* sp. RV43, ABD52_RS02395 to ABD52_RS02415; *Kitasatospora griseola* MF730-N6, TR51_RS11025 to TR51_RS11045; *Streptomyces purpeofuscus* NRRL B-1817^T^, IF01_RS0123020 to IF01_RS0123045; *Streptomyces* sp. NRRL F-6131, IF39_RS0107420 to IF39_RS0107445; *Streptomyces* sp. XY431, ADK60_02665 to ADK60_02635; *Kitasatospora* sp. MBT66, BI06_RS24475 to BI06_RS24440; *Streptomyces celluloflavas* NRRL B-2493^T^, IH09_RS02990 to IH09_RS03015; *Streptomyces albus* subsp. *albus* NRRL B-2513, ACZ90_11100 to ACZ90_11120

Since the BLAST analysis shown in Table 6 suggests that other *Micromonospora* strains such as *M. purpureochromogenes* and *Micromonospora* sp. M42 may possess *rak* clusters, hybrid PKS/NRPS gene clusters similar to *rak* clusters were searched for bacterial strains whose genome sequences and the ORF information are available in the GenBank database. We carried out BLAST search using RakEF sequence of *Micromonospora* sp. DSW705 and *Streptomyces* sp. MWW064 as the queries, and then analyzed each of the gene clusters encoding RakEF orthologues using antiSMASH [19] and manually if necessary. As shown in Fig. 4, three *Micromonospora*, 19 *Streptomyces*, three *Frankia*, one *Nocardiopisis*, one *Salinispora* *,* and two *Kitasatospora* strains were found to possess hybrid PKS/NRPS gene clusters encoding RakEF orthologues. On the basis of the domain organizations and amino-acids substrates of A domains, these gene clusters can be classified into four groups (Fig. 4).

*M. purpureochromogenes* NRRL B-2672 harbors a *rak* cluster as same as *Micromonospora* sp. DSW705 and *Streptomyces* sp. MWW064. *Micromonospora* sp. M42 also possesses almost the same cluster, but the methyltransferase (MT) domain in module 5 (m5) is not present and some ORFs are fragmented (Fig. 4a).

**Fig. 5:**
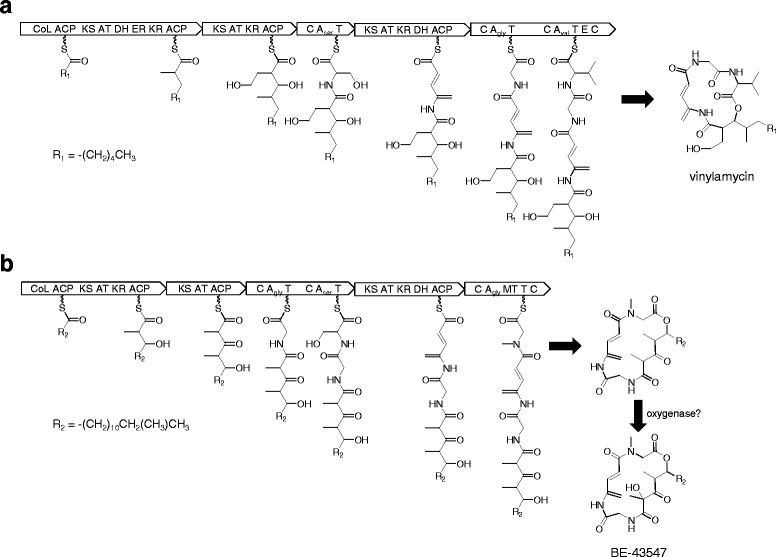
Putative biosynthetic pathways for vinylamycin (**a**) and BE-43547 (**b**)

Eighteen gene clusters categorized into Fig. 4b have domain organizations similar to *rak* clusters but the substrate of A domain in m6 was predicted to be L-valine. As vinylamycin and microtermolide contain a valine residue in their depsipeptide structure [1, 2], the four gene clusters of “*Streptomyces* *rubellomurinus*” ATCC 31215 and three *Frankia* strains were proposed to be responsible for vinylamycin biosynthesis. A plausible biosynthetic pathway for vinylamycin is illustrated in Fig. 5a. If the loading modules incorporate a C_3_ unit or LMs encode an AT domain for a C_3_ starter instead of the CoA-ligase domain, the cluster is likely responsible for microtermolide biosynthesis. The remaining 14 strains in Fig. 4b lack a KR domain in m2. In the clusters of eight among the 18 strains, NRPSs for m5 and m6 are encoded the complementary strands, although the cluster of *Streptomyces durhamensis* NRRL ISP-5539^T^ was not completely sequenced. *Streptomyces* sp. 769 does not have the PKS for LM and m1. In the cluster of *Streptomyces* sp. MspMP-M5, the PKS likely for LM and m1 is encoded downstream of the PKS gene for m4, although the gene cluster was not completely sequenced. The cluster of *Nocardiposis* sp. CNS639 likely lacks a LM, and some domains are distinct from those of other strains.

Gene clusters of *Salinispora arenicola* CNR107 and *Micromonospora* sp. RV43 contain three NRPS modules at m3, m4, and m6, which were predicted to incorporate glycine, serine, and glycine, respectively. Only BE-43547 is known as a depsipeptide containing two glycines and APDA moiety. According to the domain organization, these two clusters are proposed to be involved with BE-43547 production as illustrated in Fig. 5b.

Figure 4d shows gene clusters in which the last NRPS module incorporates amino acids different from those of the other three groups described above. Five gene clusters shown in green were predicted to incorporate L-tyrosine into the polyketide/nonribosomal peptide chains by m6. Since depsipeptides bearing both tyrosine and APDA residues are not known, products from these clusters may be structurally novel. Two gene clusters of *Streptomyces* *celluloflavas* NRRL B-2493^T^ and *Streptomyces albus* subsp. *albus* NRRL B-2513 showed the same domain organization as *rak* clusters, but NRPS substrate prediction suggests incorporation of L-glutamate and L-tryptophan/β-hydroxy-tyrosine (bht) by m6, respectively. Because rakicidin analogues containing these amino acids in place of the asparagine residue have not been reported, production of novel APDA-containing peptides is expected in these strains.

### Distribution of the gene clusters among genome-sequenced strains

**Fig. 6:**
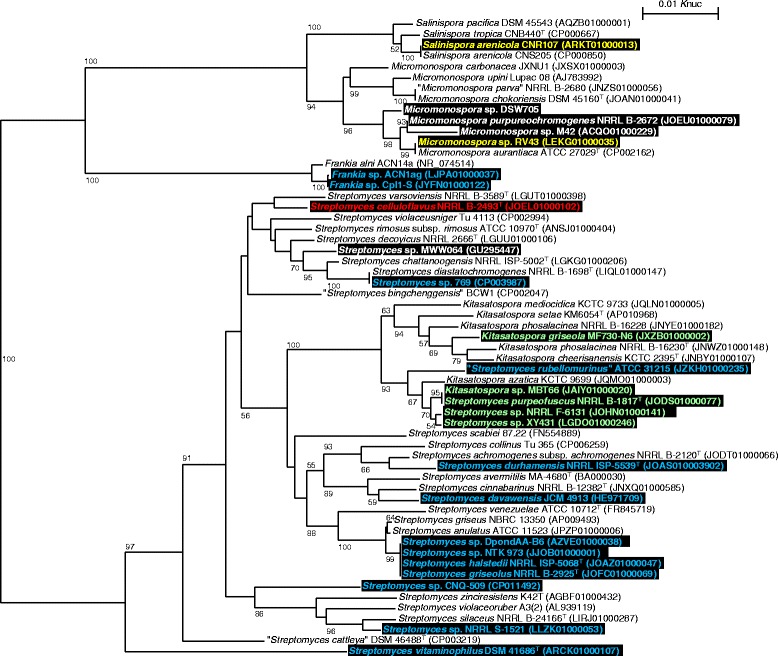
Phylogenetic tree of genome-sequenced actinomycete strains based on 16S rRNA gene sequences. Strains harboring biosynthetic gene clusters for rakicidin and the related compound are shaded in black. Strains are colored according to the group shown in Fig. 3: Fig. 3a, white; Fig. 3b, blue; Fig. 3c, yellow; Fig. 3d, green and red. Strains whose 16S rRNA gene sequences are neither registered nor almost complete are excluded from this analysis

Whole genome sequencing has been performed for a large number of actinomycete strains. At present, genome sequences of over 227 *Streptomyces* species, eight species and six strains of *Kitasatospora*, eight species and seven strains of *Micromonospora*, three *Salinispora* species, one species and 97 strains of *Frankia*, and 18 species and 6 strains of *Nocardiopsis* are available from the GenBank database. Among them, 29 strains possess the *rak*-like gene clusters. To investigate the correlation between evolution and secondary metabolite gene distribution, strains harboring the *rak*-like gene clusters (shaded in black) were mapped onto the phylogenetic tree of genome-sequenced strains based on 16S rRNA gene sequences (Fig. 6). *Micromonospora* strains are divided into two clades, one of which includes three rakicidin-producers and one BE-43547-producer. Strain MWW064 is the only *Streptomyces* that possesses the *rak* cluster other than *Micromonospora*. In contrast, vinylamycin-related gene clusters, shown in blue, are distributed in taxonomically diverse *Streptomyces* strains. It is noteworthy that two *Frankia* strains have the same gene cluster whereas only four compounds have been described for *Frankia* species [21]. This genus should be more examined for secondary metabolite production. BE-43547 gene clusters are present only in two strains of two genera belonging to the family *Micromonosporaceae* in this analysis. But, since this compound was originally found from *Streptomyces* [3], the gene cluster must also be present in the genus *Streptomyces*. Presence of gene clusters for depsipeptides containing a tyrosine residue is limited to the genus *Kitasatospora* and phylogenetically close　*Streptomyces* members. The *S. celluloflavas* NRRL B-2493^T^ gene cluster shows a similar domain organization to those of *rak* clusters stated above, but this strain is not taxonomically close to rakicidin producers.

## Conclusions

The 6.8 Mb draft genome of *Micromonospora* sp. DSW705, a producer of rakicidins A and B isolated from deep seawater, has been deposited at GenBank/ENA/DDBJ under the accession number BBVA00000000. This strain contains seven PKS and NRPS gene clusters, from which rakicidin-biosynthetic gene cluster was identified. Gene clusters for the synthesis of rakicidins or the related compounds are present in taxonomically diverse actinomycete strains, belonging to *Micromonospora*, *Salinispora*, *Frankia*, *Nocardiposis*, *Kitasatospora*, and *Streptomyces*. These findings provide useful information for discovering new and diverse depsipeptides bearing the APDA unit, and accelerate understanding of relationship between taxonomy and secondary metabolite gene distribution, and will possibly provide the insight regarding to the evolution of secondary metabolite genes.

## Abbreviations

A: Adenylation
ABC: ATP-binding cassette
ACP: Acyl carrier protein
APDA: 4-amino-2,4-pentadienoate
AT: Acyltransferase
ATP: Adenosine triposphate
bht: β-hydroxy-tyrosine
C: Condensation
CoA: Coenzyme A
CoL: CoA ligase
DDBJ: DNA Data Bank of Japan
DH: Dehydratase
E: Epimerization
ER: Enoylreductase
ISP: International *Streptomyces* project
KR: Ketoreductase
KS: Ketosynthase
LM: Loading module
m: Module
MT: Methyltransferase
NBRC: Biological Resource Center, National Institute of Technology and Evaluation
NRPS: Nonribosomal peptide synthetase
PKS: Polyketide synthase
T: Thiolation

## References

[1] Carr G, Poulsen M, Klassen JL, Hou Y, Wyche TP, Bugni TS, Currie CR, Clardy J (2012). Microtermolides A and B from termite-associated *Streptomyces* sp. and structural revision of vinylamycin. Org Lett.

[2] Igarashi M, Shida T, Sasaki Y, Kinoshita N, Naganawa H, Hamada M, Takeuchi T (1999). Vinylamycin, a new depsipeptide antibiotic, from *Streptomyces* sp. J Antibiot.

[3] Nishioka H, Nakajima S, Nagashima M, Kojiri K, Suda H (1998). BE-43547 series substances, their manufacture with *Streptomyces* species, and their use as antitumor agents. Japan Patent.

[4] Igarashi Y, Shimasaki R, Miyanaga S, Oku N, Onaka H, Sakurai H, Saiki I, Kitani S, Nihira T, Wimonsiravude W (2010). Rakicidin D, an inhibitor of tumor cell invasion from marine-derived *Streptomyces* sp. J Antibiot.

[5] McBrien KD, Berry RL, Lowe SE, Neddermann KM, Bursuker I, Huang S, Klohr SE, Leet JE (1995). Rakicidins, new cytotoxic lipopeptides from *Micromonospora* sp. fermentation, isolation and characterization. J Antibiot.

[6] Oku N, Matoba S, Yamazaki YM, Shimasaki R, Miyanaga S, Igarashi Y (2014). Complete stereochemistry and preliminary structure-activity relationship of rakicidin A, a hypoxia-selective cytotoxin from *Micromonospora* sp. J Nat Prod.

[7] Hu J, Wunderlich D, Sattler I, Feng X, Grabley S, Thiericke R (2000). Rakicidin C, a new cyclic depsipeptide from *Streptomyces* sp. European J Org Chem.

[8] Komaki H, Ichikawa N, Hosoyama A, Fujita N, Igarashi Y (2015). Draft genome sequence of marine-derived *Streptomyces* sp. TP-A0598, a producer of anti-MRSA antibiotic lydicamycins. Stand Genomic Sci.

[9] Kim OS, Cho YJ, Lee K, Yoon SH, Kim M, Na H, Park SC, Jeon YS, Lee JH, Yi H (2012). Introducing EzTaxon-e: a prokaryotic 16S rRNA gene sequence database with phylotypes that represent uncultured species. Int J Syst Evol Microbiol.

[10] Larkin MA, Blackshields G, Brown NP, Chenna R, McGettigan PA, McWilliam H, Valentin F, Wallace IM, Wilm A, Lopez R (2007). Clustal W and Clustal X version 2.0. Bioinformatics.

[11] Perriere G, Gouy M (1996). WWW-query: an on-line retrieval system for biological sequence banks. Biochimie.

[12] Kimura M (1980). A simple method for estimating evolutionary rates of base substitutions through comparative studies of nucleotide sequences. J Mol Evol.

[13] Hasegawa T, Takizawa M, Tanida S (1983). A rapid analysis for chemical grouping of aerobic actinomycetes. J Gen Appl Microbiol.

[14] Hamada M, Yamamura H, Komukai C, Tamura T, Suzuki K, Hayakawa M (2012). *Luteimicrobium album* sp. nov., a novel actinobacterium isolated from a lichen collected in Japan, and emended description of the genus *Luteimicrobium*. J Antibiot.

[15] Field D, Garrity G, Gray T, Morrison N, Selengut J, Sterk P, Tatusova T, Thomson N, Allen MJ, Angiuoli SV (2008). The minimum information about a genome sequence (MIGS) specification. Nat Biotechnol.

[16] Ohtsubo Y, Maruyama F, Mitsui H, Nagata Y, Tsuda M (2012). Complete genome sequence of *Acidovorax* sp. strain KKS102, a polychlorinated-biphenyl degrader. J Bacteriol.

[17] Hyatt D, Chen GL, Locascio PF, Land ML, Larimer FW, Hauser LJ (2010). Prodigal: prokaryotic gene recognition and translation initiation site identification. BMC Bioinformatics.

[18] Lowe TM, Eddy SR (1997). tRNAscan-SE: a program for improved detection of transfer RNA genes in genomic sequence. Nucleic Acids Res.

[19] Blin K, Medema MH, Kazempour D, Fischbach MA, Breitling R, Takano E, Weber T (2013). antiSMASH 2.0--a versatile platform for genome mining of secondary metabolite producers. Nucleic Acids Res.

[20] Fischbach MA, Walsh CT (2006). Assembly-line enzymology for polyketide and nonribosomal peptide antibiotics: logic, machinery, and mechanisms. Chem Rev.

[21] CRCnetBase. Dictionary of Natural Products on DVD-ROM, version 19:1. London: Chapman & Hall; 2014.

[22] Woese CR, Kandler O, Wheelis ML (1990). Towards a natural system of organisms: proposal for the domains *Archaea*, *Bacteria*, and *Eucarya*. Proc Natl Acad Sci U S A.

[23] Goodfellow M, Goodfellow M, Kämpfer P, Busse H-J, Trujillo ME, Suzuki K-I, Ludwig W, Whitman WB (2012). Phylum XXVI. *Actinobacteria* phyl. nov. Bergey's Manual of Systematic Bacteriology, Second Edition, Volume 5, Part A.

[24] Stackebrandt E, Rainey FA, Ward-Rainey NL (1997). Proposal for a new hierarchic classification system, *Actinobacteria* classis nov. Int J Syst Bacteriol.

[25] Buchanan RE (1917). Studies in the Nomenclature and Classification of the Bacteria: II. The Primary Subdivisions of the *Schizomycetes*. J Bacteriol.

[26] Skerman VBD, McGowan V, Sneath PHA (1980). Approved lists of bacterial names. Int J Syst Bacteriol.

[27] Zhi XY, Li WJ, Stackebrandt E (2009). An update of the structure and 16S rRNA gene sequence-based definition of higher ranks of the class *Actinobacteria*, with the proposal of two new suborders and four new families and emended descriptions of the existing higher taxa. Int J Syst Evol Microbiol.

[28] Koch C, Kroppenstedt RM, Rainey FA, Stackebrandt E (1996). 16S ribosomal DNA analysis of the genera *Micromonospora*, *Actinoplanes*, *Catellatospora*, *Catenuloplanes*, *Couchioplanes*, *Dactylosporangium*, and *Pilimelia* and emendation of the family *Micromonosporaceae*. Int J Syst Bacteriol.

[29] Krasil'nikov NA (1938). Ray fungi and related organisms. *Actinomycetales*, Akademii Nauk, Moscow.

[30] Ørskov J (1923). Investigations into the morphology of the ray fungi.

[31] Ashburner M, Ball CA, Blake JA, Botstein D, Butler H, Cherry JM, Davis AP, Dolinski K, Dwight SS, Eppig JT (2000). Gene ontology: tool for the unification of biology. The Gene Ontology Consortium. Nat Genet.

[32] Kakavas SJ, Katz L, Stassi D (1997). Identification and characterization of the niddamycin polyketide synthase genes from *Streptomyces caelestis*. J Bacteriol.

[33] Saitou N, Nei M (1987). The neighbor-joining method: a new method for reconstructing phylogenetic trees. Mol Biol Evol.

